# On the Use of Chloroform Ointment in Rigidity of the Os Uteri

**Published:** 1854-05

**Authors:** G. W. Ronald

**Affiliations:** Physician to the Louisville Alms-House


### Art. II.—ON THE USE OF CHLOROFORM OINTMENT IN RIGIDITY OF THE OS UTERI.

BY G. W. RONALD, M. D., PHYSICIAN TO THE LOUISVILLE ALMS-HOUSE.

There are with few exceptions, no accidents to which the parturient woman is subject that inflict upon her a greater amount of suffering than obstinate rigidity of the os uteri. It not unfrequently baffles the skill of the oldest and most experienced practitioner for a long time; and were we to judge from the pain dependent upon this condition of the organ, we should very readily suppose that the life of our patient was in the most imminent danger. This state of affairs as compared with the number of births, is fortunately of rare occurrence; yet it is of sufficient frequency to make the subject one of considerable interest and importance in practice. Various causes have been assigned, by different authors, for rigidity of the os uteri; some attributing it to mechanical contrivances used for supporting the organ, others to the injudicious use of escharotics, whilst others again contend that morbid irritability is the principal cause, and this last conclusion seems to us more rational than either of the others. It is not my intention, however, to enter into an inquiry as to the cause of the difficulty; but to call the attention of the profession to the use of chloroform ointment, as I have found it the most expeditious, reliable, and safe mode of overcoming it. I do not pretend to say that it is a specific, for my limited experience in its use would not justify such a conclusion, having used it in but a few instances, but in these, with the most gratifying results. The first time that I made a trial of it was in the Louisville Almshouse, on the 28th day of January, 1852.

Mrs. C., aged twenty-three, a seamstress, was admitted on the 25th, reputed to be of respectable family, full and plethoric habits, nine months advanced in pregnancy with her first child. She was complaining of pain in the left side—considerable œdema of the lower extremities—bowels constipated, for which oil had been repeatedly taken, urine highly colored and scanty. Ordered a Seidlitz powder to be given, and as soon as it operated to be followed with a teaspoonfull of sweet spirits of nitre every two hours. At 7 o'clock on the morning of the 28th, she was taken in labor. I saw her at 10 o'clock. Upon making an examination, the os uteri was sufficiently dilated to ascertain a head presentation, though the membranes were not ruptured. The pains were powerful and strong, continued to return at short and regular intervals, and I consoled myself with the thought that I should be detained but a short time, yet hour after hour passed and still the os uteri had made no perceptible progress towards dilatation. The woman had become restless and despondent, intense thirst, sickness at the stomach with constant retching, throwing off the water almost as soon as swallowed. If the os uteri was touched she complained of pain, it was hot and unusually rigid, feeling as if a tight band or cord had been drawn around the neck of the organ, which was resisting and unyielding. Having waited upon nature to overcome the difficulty until the patience of the woman, as well as of her attendants was completely exhausted, I determined to resort to some of the remedies usually recommended. Accordingly the arm was tied, and blood abstracted to approaching syncope. Tartrate of antimony, and the warm bath, all in their turn were brought into requisition; yet the condition of the organ had changed but little, though the membranes at this time had given way and discharged a portion of liquor amnii. These means having failed to procure the desired effect, I went to the office for the purpose of making an ointment of belladonna, but was disappointed in finding none in the house. When I was in the act of sending to town for the article it occurred to me that the ointment of chloroform might as readily relieve rigidity of the os uteri, as contraction of the muscles of the extremities, which I had often seen it do, having had it applied to my own person in an attack of cholera, by my friend and preceptor, Dr. T. S. BELL. Taking this view of the subject, I determined to try it, though not without some doubts and apprehensions, for I knew not what effect it might have upon the child, or upon the hot and irritable os uteri. The ointment was prepared by taking one drachm and a half of chloroform and thoroughly incorporating it with one ounce of simple cerate; which was freely applied principally upon the external surface of the neck of the organ. At the time of the application the woman complained of slight smarting pain which passed off in the course of a few minutes, and had it been applied at the commencement, or during contraction of the organ, she would have made no complaint, as was fully proved in the subsequent cases in which I used it. Upon making an examination, twenty-five minutes after the application of the ointment, I was surprised to find the os uteri dilating rapidly, soft and pliant; and in one hour and twenty-seven minutes after its first application the woman was delivered of a fine, robust and healthy boy.

I am well aware that any manual interference on the part of the practitioner, in order to terminate protracted or lingering labor, is condemned by some of the ancient writers upon obstetrics. Dr. BLUNDELL, in his valuable and scientific work reminds us in every chapter that "meddlesome midwifery is bad." This I suppose no one will doubt. But surely no well informed physician, in this age of improvement and advancement, both in the arts and sciences, when every thing seems to be moving on with almost telegraphic speed, will condemn the use of any remedy that we may have at our disposal for shortening the pangs of one of the most painful processes in the parturient woman.

